# MetAssimulo 2.0: a web app for simulating realistic 1D and 2D metabolomic ^1^H NMR spectra

**DOI:** 10.1093/bioinformatics/btaf045

**Published:** 2025-01-25

**Authors:** Yan Yan, Beatriz Jiménez, Michael T Judge, Toby Athersuch, Maria De Iorio, Timothy M D Ebbels

**Affiliations:** Section of Bioinformatics, Division of Systems Medicine, Department of Metabolism, Digestion and Reproduction, Faculty of Medicine, Imperial College London, London W12 0NN, United Kingdom; National Phenome Centre & Section of Bioanalytical Chemistry, Department of Metabolism, Digestion and Reproduction, Imperial College London, London W12 0NN, United Kingdom; Section of Bioinformatics, Division of Systems Medicine, Department of Metabolism, Digestion and Reproduction, Faculty of Medicine, Imperial College London, London W12 0NN, United Kingdom; Institute for Bioscience and Biotechnology Research, University of Maryland, Rockville, MD 20850, United States; Section of Bioinformatics, Division of Systems Medicine, Department of Metabolism, Digestion and Reproduction, Faculty of Medicine, Imperial College London, London W12 0NN, United Kingdom; Yong Loo Lin School of Medicine, National University of Singapore, Singapore 117597, Singapore; A*STAR Institute for Human Development and Potential, Singapore 117609, Singapore; Section of Bioinformatics, Division of Systems Medicine, Department of Metabolism, Digestion and Reproduction, Faculty of Medicine, Imperial College London, London W12 0NN, United Kingdom

## Abstract

**Motivation:**

Metabolomics extensively utilizes nuclear magnetic resonance (NMR) spectroscopy due to its excellent reproducibility and high throughput. Both 1D and 2D NMR spectra provide crucial information for metabolite annotation and quantification, yet present complex overlapping patterns which may require sophisticated machine learning algorithms to decipher. Unfortunately, the limited availability of labeled spectra can hamper application of machine learning, especially deep learning algorithms which require large amounts of labeled data. In this context, simulation of spectral data becomes a tractable solution for algorithm development.

**Results:**

Here, we introduce MetAssimulo 2.0, a comprehensive upgrade of the MetAssimulo 1.b metabolomic ^1^H NMR simulation tool, reimplemented as a Python-based web application. Where MetAssimulo 1.0 only simulated 1D ^1^H spectra of human urine, MetAssimulo 2.0 expands functionality to urine, blood, and cerebral spinal fluid, enhancing the realism of blood spectra by incorporating a broad protein background. This enhancement enables a closer approximation to real blood spectra, achieving a Pearson correlation of approximately 0.82. Moreover, this tool now includes simulation capabilities for 2D *J*-resolved (*J*-Res) and Correlation Spectroscopy spectra, significantly broadening its utility in complex mixture analysis. MetAssimulo 2.0 simulates both single, and groups, of spectra with both discrete (case–control, e.g. heart transplant versus healthy) and continuous (e.g. body mass index) outcomes and includes inter-metabolite correlations. It thus supports a range of experimental designs and demonstrating associations between metabolite profiles and biomedical responses.

By enhancing NMR spectral simulations, MetAssimulo 2.0 is well positioned to support and enhance research at the intersection of deep learning and metabolomics.

**Availability and implementation:**

The code and the detailed instruction/tutorial for MetAssimulo 2.0 is available at https://github.com/yanyan5420/MetAssimulo_2.git. The relevant NMR spectra for metabolites are deposited in MetaboLights with accession number MTBLS12081.

## 1 Introduction

Metabolomics, an essential part of systems biology, provides an exhaustive profile of metabolite variations in response to genetic and external changes (Nicholson *et al.* 1999, Beckonert *et al.* 2007). Proton nuclear magnetic resonance (^1^H NMR) spectroscopy, known for its reproducibility and nondestructive nature, has become a pivotal technique in metabolic profiling, offering rich chemical structural insights (Beckonert *et al.* 2007). The utility of both 1D and 2D NMR spectra in the metabolite identification is well documented (Rossé *et al.* 2002, Cobas *et al.* 2013, Dona *et al.* 2016, Marchand *et al.* 2017, Judge and Ebbels 2022). Moreover, the integration of sophisticated machine learning algorithms has paved the way for extracting meaningful biological insights from intricate NMR spectroscopic datasets. Nevertheless, the scarcity of NMR spectral data poses a challenge to the efficacy of these algorithms and the validation of the biological significance (Muncey *et al.* 2010). Recently it was demonstrated that simulated NMR data could be used to train deep learning algorithms, emphasizing its critical role in addressing the challenges posed by data scarcity (Yan *et al.* 2024).

To address this limitation, synthetic NMR spectra simulation has been explored. Notably, Muncey *et al.* (2010) introduced MetAssimulo 1.0, a MATLAB-based package for simulating realistic pH-dependent ^1^H NMR spectra for complex mixtures such as urine in 2010. This was followed by Atieh *et al.* (2013), who developed MetFlexo, a C package enabling the simulation of ^1^H NMR spectra without constraints on pH or magnetic field strength. Dashti *et al.* (2017) designed a web tool named GiSSMO, which simulates 1D ^1^H NMR spectra of metabolites across different field strengths and facilitates the generation of simple mixture spectra. Despite these advancements, the installation requirements of the existing tools limit their accessibility. More importantly, the simulation of 2D NMR spectra for complex mixtures remains underexplored.

Here, we introduce MetAssimulo 2.0, an advanced reimplementation and expansion of MetAssimulo 1.0 (Muncey *et al.* 2010), now presented as a user-friendly web application developed in Python. MetAssimulo 2.0 is designed to enhance functionality and accessibility, supporting both discrete outcomes comparing mixture spectra under varying conditions (e.g. “normal” versus “abnormal”) and continuous outcomes demonstrating associations between metabolites and biomedical responses, such as age or body mass index (BMI). MetAssimulo 2.0 adds blood and cerebral spinal fluid (CSF) as alternative biofluids and enhances the realism for blood by incorporating a broad protein background a key feature of real blood spectra. Simulation of urine spectra benefits from MetAssimulo 1.0’s ability to shift metabolite resonances according to pH, and extends its capability to the simulation of 2D J-resolved (J-Res) and Correlation Spectroscopy (COSY) NMR spectra for complex mixtures without pH limitations.

## 2 Methods

The core algorithm of MetAssimulo 2.0 generates simulated NMR spectra for mixtures by employing a linear combination of experimental ^1^H NMR spectra from pure compounds (Muncey *et al.* 2010):


(1) $$y\left(\delta \right)=\sum_{k=1}^{K}c_{k}p_{k}\gamma \left(\delta \right)$$

where *y*(*δ*) denotes the signal intensity at chemical shift *δ* ppm for a mixture of *K* metabolites *k = 1,…,K*, each of which is present at concentration *c_k_* and contains *p_k_* observable protons.

### 2.1 Pure compound reference spectra database

To construct the local database, the most commonly found metabolites in urine, blood, and CSF were selected based on concentration data extracted from the Human Metabolome Database (HMDB) version 5.0 (Wishart *et al.* 2022). The selection criteria were set to “Normal,” “Adults,” and “Both” for sex. Detailed information on the selected pure compounds is provided in Supplementary Table S1. The spectral data for these metabolites were obtained from the National Phenome Centre at Imperial College London (see experimental procedures in Supplementary Material S1.2).

### 2.2 Determination of metabolite concentrations in simulated biofluids

#### 2.2.1 Discrete outcomes

MetAssimulo 2.0 expands the functionality of MetAssimulo 1.0 by integrating a more comprehensive set of concentration data from HMDB 5.0 (Wishart *et al.* 2022). For metabolites with multiple entries that meet the filtering criteria, we calculate the mean ($\bar{c}$) and SD ($\overset{-}{\sigma }$) of their concentrations by averaging across all relevant sources. Specifically, when concentration data are provided as a range, we estimate the mean and SD assuming the minimum and maximum values correspond to a 99.7% (3*σ*) confidence interval for a normal distribution. Concentrations for each metabolite are then sampled from a truncated normal distribution using these parameters, following the methodology outlined in MetAssimulo 1.0 (Muncey *et al.* 2010).

As an example of abnormal conditions, “heart transplant” was chosen as a case study (these data were more readily accessible in HMDB 5.0). For other abnormal conditions, users are required to input or upload their own concentration data, which allows for customization by the user in terms of the fold change and SD ratio with respect to the normal group.

#### 2.2.2 Continuous outcomes

While MetAssimulo 1.0 only allowed for a discrete two-class outcome, MetAssimulo 2.0 introduces the capability to simulate a set of metabolites correlating with a continuous biological variable, such as age or BMI. The approach assumes a linear relationship between the metabolites (*x*) and the biological response (y), represented by a simple linear regression model. To accommodate different scales among variables, standardization is applied, transforming the model to equation (2).


(2) $$\frac{y-\bar{y}}{\sigma_{y}}=a\frac{x-\bar{x}}{\sigma_{x}}+\varepsilon_{x}$$

In this framework, users are required to input the mean concentration ($\bar{x}$) and SD ($\sigma_{x}$) for each metabolite, along with the Pearson correlation coefficient “a” and the SD of the random error $“\varepsilon_{x}”$. By default, both a and $\varepsilon_{x}$ are set to zero, indicating no assumed correlation between the metabolites and the biological response (y).

Additionally, users are required to specify the number of replicates (*n*) and the distribution of the biological response (y) by setting its mean and SD. Using these inputs, MetAssimulo 2.0 samples n values to calculate the sample mean and sample SD for each metabolite and the response. This process allows for the computation of simulated concentrations for each metabolite based on equation (2). As with discrete outcomes, users have the option to input or upload their own concentration data for completely customized simulations.

#### 2.2.3 Intra- and intermetabolite correlations

In both discrete and continuous outcome simulations, MetAssimulo 2.0 maintains the functionality of intra- and intermetabolite correlations as established in the original MetAssimulo (Muncey *et al.* 2010), ensuring that the relational dynamics between metabolites are accurately and reliably modeled. This functionality is detailed in Supplementary Material S1.3.

### 2.3 Simulated spectra for metabolite mixtures

Before simulating spectra for mixtures, it is crucial to preprocess the spectra of pure compounds to ensure they integrate effectively into the final metabolic profiles. The details for peak preprocessing can be found in Supplementary Materials S1.4 and S1.5.

#### 2.3.1 Peak detection

Peak detection is a crucial step in the analysis of pure compound spectra, particularly essential for locating and shifting peaks accurately. In the case of 1D ^1^H spectra, MetAssimulo 2.0 introduces a more flexible and efficient peak detection method which involves locating peaks based on a user-defined intensity threshold, detecting and marking peak clusters by their intensity, and filtering out clusters without marked peaks. The approach used in MetAssimulo 1.0, depended on cross-referencing detected peaks with HMDB multiplet data which could be problematic when multiplet data are lacking in HMDB (see more details in Supplementary Material S1.6).

The peak detection method for 2D J-Res and COSY spectra aligns with the procedures established for 1D ^1^H spectra, with an additional preliminary step. For 2D *J*-Res spectra, a skyline projection (Fonville *et al.* 2010) along the chemical shift (F2) axis is conducted to obtain a projected 1D profile. Similarly, in COSY spectra, a diagonal projection is employed. These projections serve as inputs for Algorithm 2, as detailed in the Supplementary Material S1.6, facilitating the localization of peak clusters in the projected profiles of 2D spectral data.

#### 2.3.2 Peak shift

In MetAssimulo 2.0, the approach for peak shifting in 1D ^1^H spectra adheres closely to the method established in MetAssimulo 1.0, incorporating pH variation as the primary factor for peak shifts. This process is quantified in equation (3), a transformation of the Henderson–Hasselbalch equation (Ackerman *et al.* 1996), tailored to address the pH-related shifts in NMR spectra (see the detailed derivation of the transformation in Supplementary Material S1.7).


(3) $$\Delta \delta =\frac{\left(\delta_{L}-\delta_{HL}\right)\times \left(10^{{pH}_{0}-\mathrm{pKa}}-10^{{pH}_{1}-\mathrm{pKa}}\right)}{\left(1+10^{{pH}_{0}-\mathrm{pKa}}\right)\times \left(1+10^{{pH}_{1}-\mathrm{pKa}}\right)}$$

where Δδ is the amount the peak is shifted in ppm; $\delta_{L}$ and $\delta_{HL}$ are the positions of the peak in the acid and basic limits (ppm), respectively; ${pH}_{0}$ is the pH of the standard spectrum, usually as 7.4; ${pH}_{1}$ is the pH of the sample; pKa is the pKa of the metabolite.

To facilitate this peak shifting, users are required to input the mean and SD of the pH values for their samples. Additionally, pKa values are typically retrieved from HMDB 5.0 (Wishart *et al.* 2022). In instances where pKa values are not available in HMDB, they are estimated by sampling from a normal distribution, with parameters (mean and SD) derived from Tredwell *et al.* (2016). This approach also extends to determining the mean and SD of the differences between acidic and basic limits, ensuring a comprehensive and precise shifting of NMR spectral peaks.

The peak shifting approach for 2D J-Res and COSY data parallels that of 1D spectral data. After peak clusters are detected on the projected profiles, the shifts are calculated using equation (3), in the same manner as 1D spectra. The detailed shifting process can be found in Supplementary Material S1.8.

## 3 Results

MetAssimulo 2.0 introduces advanced data simulation capabilities across three of the most common NMR spectra using in metabolomics: 1D, 2D J-Res, and 2D COSY. Each simulation involves three primary steps: selecting metabolites, simulating concentrations, and simulating final spectra. The detailed workflow for these processes is illustrated in Supplementary Material S2.1. This section presents case studies that utilize the key functionalities distinguishing MetAssimulo 2.0 from its predecessor, showcasing the advancements in simulation capabilities. For additional capabilities, including simulations with peak shifts and simulations of 2D COSY data without peak shifts, further details are available in Supplementary Figs S2 and S3.

### 3.1 1D simulation

#### 3.1.1 Simulation of blood mixtures with protein background

**Figure 1.**
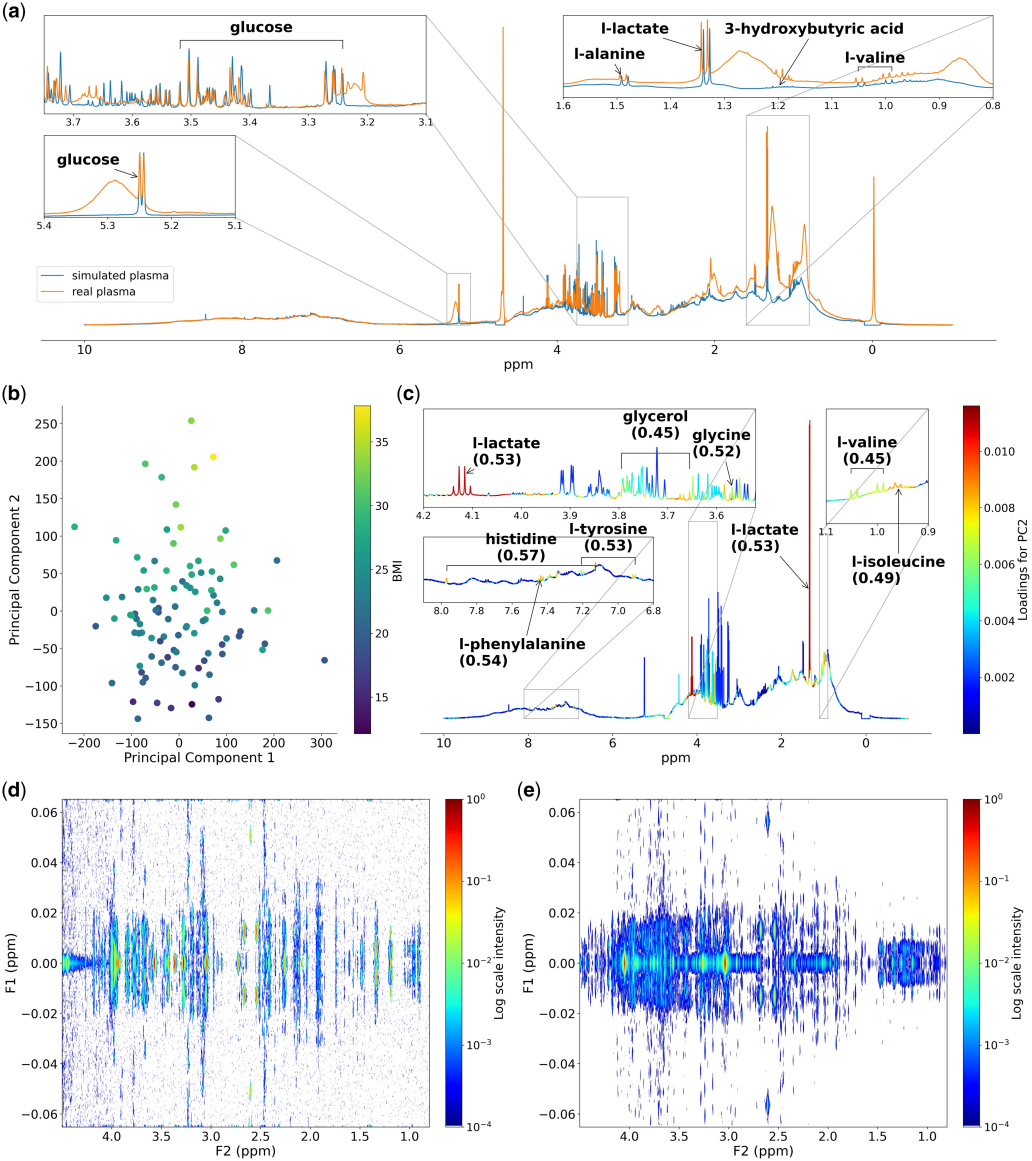
(a) Comparison between the simulated and real 1D ^1^H NMR spectrum for blood. (b) PCA score plot of the first two principal components derived from the simulated spectra, colored by the BMI values. (c) Average spectrum of 100 replicates colored by the loadings on PC2; Pearson correlation coefficients between the metabolite concentration and BMI are shown in brackets. (d) Simulated 2D J-Res urine spectrum. (e) Real 2D J-Res urine spectrum.

Figure 1a illustrates the comparison between a simulated blood spectrum with protein background (blue) and the real blood spectrum (orange), calibrated to the glucose peak at 5.23 ppm. The addition of the albumin spectrum to the simulated blood general profile provides a very realistic picture as shown for the good overlap with the real blood spectrum. The main remaining discrepancy is the macromolecule profile in regions where lipoprotein signals are observed such as from 0 to 2 ppm. Despite these differences, the simulation effectively captures both high-intensity signals, such as lactate, and low-intensity signals like alanine, 3-hydroxybutyric acid, and valine, illustrating the spectrum’s realistic representation across a range of metabolite concentrations.

#### 3.1.2 Simulation of continuous outcomes

To explore the feature of simulating continuous outcomes, we examined how metabolites are associated with BMI. Based on Moore *et al.* (2014), we identified 10 metabolites correlated with BMI: valine, isoleucine, tyrosine, leucine, glycerol, phenylalanine, lactate, asparagine, glycine, and histidine. Simulations were run for 100 replicates of blood mixtures containing these metabolites, without peak shifts. The statistical parameters including means, SD, and the correlation coefficients with BMI for these metabolites are detailed in Supplementary Table S2.

Subsequent principal component analysis (PCA) on the simulated spectra assessed how variations in these metabolites correlate with BMI. As shown in Fig. 1b, the second principal component (PC2) captures most of the BMI-related variance. The average spectrum from the 100 replicates, colored by loading scores on PC2, is depicted in Fig. 1c. Here, lactate exhibits the highest loading on PC2, highlighting its strong correlation with BMI. Other metabolites, including isoleucine, tyrosine, and histidine, also show notable loadings, underscoring their relationship to BMI. Such a simulation could provide a robust dataset for training machine learning algorithms in metabolic and epidemiological studies aimed at identifying metabolites associated with disease, or to calculate statistical power, for example.

### 3.2 2D simulation

#### 3.2.1 Simulation of urine mixtures in J-Res

Figure 1d shows a simulated *J*-Res spectrum of human urine, compared with a real *J*-Res spectrum in Fig. 1e. The comparison (0.8–4.5 ppm; full spectra can be found in Supplementary Fig. S4) reveals that the simulated spectrum, although not as densely populated as the real spectrum, still accurately reflects the spectral features of high and medium abundance metabolites such as creatinine and citric acid. The difference in density is due to the simulated spectrum containing 52 metabolites, whereas the real urine spectrum comprises more metabolites including many at lower levels. Despite this, both spectra display some considerable similarities, illustrating the effectiveness of the simulation in capturing key urinary metabolites. An arbitrary level of realism could be achieved by expanding the library to include more metabolites.

## 4 Conclusion

MetAssimulo 2.0 significantly enhances the simulation of NMR metabolic profile spectra, supporting a diverse range of experiments across three different biofluids and both 1D and 2D NMR techniques (Supplementary Table S3 summarizes differences between MetAssimulo 2.0 and MetAssimulo 1.0). This advancement opens new avenues for future developments in spectral data analysis, presenting extensive opportunities for research in metabolomics.

## Supplementary Material

btaf045_Supplementary_Data

## Data Availability

The code and the detailed instruction/tutorial for MetAssimulo 2.0 is available at https://github.com/yanyan5420/MetAssimulo_2.git. The relevant NMR spectra for metabolites are deposited in MetaboLights with accession number MTBLS12081.
