# Supplementary material for: MetAssimulo 2.0: a web app for simulating realistic 1D and 2D metabolomic 1H NMR spectra
Source: Bioinformatics. 2025 Jan 25;41(3):btaf045. doi: 10.1093/bioinformatics/btaf045 (PMC11889449; doi:10.1093/bioinformatics/btaf045)
Supplement: btaf045_Supplementary_Data [file btaf045_supplementary_data.docx]

**Supplementary Materials**

**MetAssimulo 2.0: a web app for simulating realistic 1D & 2D Metabolomic ^1^H NMR spectra**

Yan Yan^1^, Beatriz Jiménez^2^, Michael T. Judge^1,3^, Toby Athersuch^1*^, Maria De Iorio^4,5^ and Timothy M. D. Ebbels^1*^

^1^Section of Bioinformatics, Division of Systems Medicine, Department of Metabolism, Digestion and Reproduction, Faculty of Medicine, Imperial College London, London, W12 0NN, UK.

^2^National Phenome Centre & Section of Bioanalytical Chemistry, Department of Metabolism, Digestion and Reproduction, Imperial College London, IRDB Building, Hammersmith Campus, London, W12 0NN, UK.

^3^Institute for Bioscience and Biotechnology Research, University of Maryland, Rockville, Maryland 20850, United States.

^4^Yong Loo Lin School of Medicine, National University of Singapore, Singapore

^5^A*STAR Institute for Human Development and Potential

1. **Methods**
   1. **Table for pure compounds**

**Table S1.** Concentrations and Availability of Metabolites Across Biofluids. This table lists common metabolites identified in human urine, blood, and cerebrospinal fluid (CSF), including their HMDB IDs and mean concentration (units in uM) values (“/” in the table means the information on the concentration of metabolites is lacking, so it is not used to simulate the corresponding biological fluid) sourced from the Human Metabolome Database (HMDB) (Wishart et al. 2022). The availability of these metabolites for different NMR experiments – 1D, 2D J-Resolved (J-Res) and 2D Correlation Spectroscopy (COSY) in the Pure Compound Reference Spectra Database – is also indicated. Metabolites are ordered by decreasing concentration in urine.

| HMDB ID | Metabolite Names | Mean ($\overline{\boldsymbol{c}}$)  in Urine | Mean ($\overline{\boldsymbol{c}}$) in Blood | Mean ($\overline{\boldsymbol{c}}$) in CSF | 1D | J-Res | COSY |
| --- | --- | --- | --- | --- | --- | --- | --- |
| HMDB0000562 | creatinine | 11995.25 | 85.20 | 57.72 | ✓ | ✓ |  |
| HMDB0000714 | hippuric acid | 3207.51 | 11.41 | ／ | ✓ | ✓ | ✓ |
| HMDB0000094 | citric acid | 3038.13 | 145.90 | 257.00 | ✓ | ✓ | ✓ |
| HMDB0000064 | creatine | 1890.39 | 49.71 | 44.00 | ✓ | ✓ |  |
| HMDB0000123 | glycine | 1751.35 | 275.95 | 6.66 | ✓ | ✓ |  |
| HMDB0000925 | trimethylamine n-oxide | 1257.70 | 38.30 | ／ | ✓ | ✓ |  |
| HMDB0006372 | l-glyceric acid | 851.06 | 2.00 | ／ | ✓ | ✓ |  |
| HMDB0000251 | taurine | 786.47 | 89.75 | 6.90 | ✓ | ✓ |  |
| HMDB0000177 | l-histidine | 711.46 | 99.85 | 11.40 | ✓ | ✓ |  |
| HMDB0000190 | l-lactate | 613.78 | 2011.80 | 1817.75 | ✓ | ✓ |  |
| HMDB0006344 | phenylacetylglutamine | 607.88 | 3.34 | ／ | ✓ | ✓ | ✓ |
| HMDB0001858 | p-cresol | 591.37 | 5.36 | ／ | ✓ | ✓ |  |
| HMDB0002994 | erythritol | 560.18 | 4.10 | 37.00 |  |  |  |
| HMDB0000574 | l-cysteine | 550.35 | 74.47 | ／ | ✓ | ✓ |  |
| HMDB0000142 | formic acid | 510.86 | 103.17 | 32.00 | ✓ | ✓ |  |
| HMDB0000115 | glycolic acid | 503.80 | 7.85 | ／ | ✓ | ✓ |  |
| HMDB0000001 | 1-methylhistidine | 496.04 | 13.10 | 4.00 |  |  |  |
| HMDB0001875 | methanol | 443.82 | 77.40 | 44.00 |  |  |  |
| HMDB0000193 | isocitric acid | 427.34 | 6.00 | ／ | ✓ | ✓ | ✓ |
| HMDB0000122 | d-glucose | 412.06 | 4908.19 | 2340.00 | ✓ | ✓ |  |
| HMDB0000641 | l-glutamine | 401.17 | 578.38 | 501.00 | ✓ | ✓ | ✓ |
| HMDB0000128 | guanidoacetic acid | 399.14 | 3.98 | 0.05 |  |  |  |
| HMDB0000875 | trigonelline | 373.05 | ／ | ／ | ✓ | ✓ |  |
| HMDB0000043 | betaine | 366.45 | 63.56 | ／ | ✓ | ✓ |  |
| HMDB0000211 | myo-inositol | 351.06 | 25.67 | 132.33 |  |  |  |
| HMDB0000042 | acetic acid | 349.84 | 46.74 | 87.00 |  |  |  |
| HMDB0000187 | l-serine | 330.73 | 149.01 | 27.40 | ✓ | ✓ | ✓ |
| HMDB0000149 | ethanolamine | 325.23 | 1.60 | 11.27 | ✓ | ✓ | ✓ |
| HMDB0000087 | dimethylamine | 323.87 | 23.95 | 2.00 | ✓ | ✓ |  |
| HMDB0001851 | l-arabitol | 306.36 | 2.00 | 28.90 | ✓ | ✓ | ✓ |
| HMDB0000161 | l-alanine | 298.23 | 421.16 | 25.11 | ✓ | ✓ | ✓ |
| HMDB0000060 | acetoacetic acid | 264.50 | 35.59 | 19.10 |  |  |  |
| HMDB0000167 | l-threonine | 264.09 | 147.70 | 25.01 | ✓ | ✓ |  |
| HMDB0000765 | mannitol | 255.86 | 34.00 | 5.00 | ✓ | ✓ | ✓ |
| HMDB0000718 | isovaleric acid | 254.30 | 1.60 | ／ | ✓ | ✓ | ✓ |
| HMDB0000954 | ferulic acid | 246.62 | 0.04 | ／ | ✓ | ✓ |  |
| HMDB0000182 | l-lysine | 229.45 | 228.33 | 22.80 | ✓ | ✓ |  |
| HMDB0000568 | d-arabitol | 227.91 | ／ | 19.00 | ✓ | ✓ | ✓ |
| HMDB0000267 | pyroglutamic acid | 213.12 | 19.50 | 47.00 |  |  |  |
| HMDB0000020 | p-hydroxyphenylacetic acid | 195.74 | 0.23 | ／ | ✓ | ✓ |  |
| HMDB0000044 | ascorbic acid | 180.82 | 42.51 | 125.33 | ✓ | ✓ |  |
| HMDB0000072 | cis-aconitic acid | 176.73 | ／ | 20.00 | ✓ | ✓ |  |
| HMDB0001659 | acetone | 176.18 | 68.58 | 68.03 |  |  |  |
| HMDB0000039 | butyric acid | 174.85 | 1.00 | ／ | ✓ | ✓ |  |
| HMDB0000462 | allantoin | 163.86 | 2.10 | 9.30 | ✓ | ✓ |  |
| HMDB0000168 | l-asparagine | 154.16 | 51.56 | 66.03 | ✓ | ✓ |  |
| HMDB0000243 | pyruvic acid | 153.24 | 72.08 | 107.00 | ✓ | ✓ |  |
| HMDB0002243 | picolinic acid | 152.10 | 0.30 | 0.31 |  |  |  |
| HMDB0000158 | l-tyrosine | 147.34 | 83.43 | 9.13 | ✓ | ✓ |  |
| HMDB0000192 | l-cystine | 141.75 | 62.90 | 0.20 | ✓ | ✓ |  |
| HMDB0000011 | 3-hydroxybutyric acid | 137.27 | 65.93 | 104.10 | ✓ | ✓ | ✓ |
| HMDB0000300 | uracil | 135.58 | 1.14 | 0.10 |  |  |  |
| HMDB0000646 | l-arabinose | 123.35 | 2.50 | 14.15 |  |  |  |
| HMDB0000904 | citrulline | 117.67 | 32.60 | 3.31 | ✓ | ✓ |  |
| HMDB0000929 | l-tryptophan | 99.24 | 65.49 | 8.11 | ✓ | ✓ | ✓ |
| HMDB0000191 | l-aspartic acid | 94.88 | 13.72 | 1.33 | ✓ | ✓ | ✓ |
| HMDB0000230 | n-acetylneuraminic acid | 83.97 | 0.57 | 11.90 |  |  |  |
| HMDB0001881 | propylene glycol | 80.37 | 8.93 | 33.00 |  |  |  |
| HMDB0000159 | l-phenylalanine | 80.18 | 77.73 | 9.05 | ✓ | ✓ | ✓ |
| HMDB0000208 | oxoglutaric acid | 79.20 | 7.95 | 5.45 |  |  |  |
| HMDB0000143 | d-galactose | 78.37 | 88.30 | 166.00 | ✓ | ✓ |  |
| HMDB0004983 | dimethyl sulfone | 75.57 | 8.80 | 2.00 |  |  |  |
| HMDB0000254 | succinic acid | 72.06 | 11.20 | 15.75 | ✓ | ✓ |  |
| HMDB0000687 | l-leucine | 69.75 | 124.20 | 10.10 | ✓ | ✓ | ✓ |
| HMDB0000247 | sorbitol | 69.17 | 7.05 | 16.30 | ✓ | ✓ | ✓ |
| HMDB0001539 | asymmetric dimethylarginine | 68.18 | 198.22 | 0.06 |  |  |  |
| HMDB0000148 | l-glutamic acid | 66.42 | 55.22 | 10.72 | ✓ | ✓ | ✓ |
| HMDB0002183 | docosahexaenoic acid | 65.97 | 68.23 | 0.15 |  |  |  |
| HMDB0000906 | trimethylamine | 61.18 | 13.49 | 0.07 | ✓ | ✓ |  |
| HMDB0000699 | 1-methylnicotinamide | 57.60 | 0.43 | ／ | ✓ | ✓ | ✓ |
| HMDB0000062 | l-carnitine | 56.98 | 39.81 | 2.95 |  |  |  |
| HMDB0000157 | hypoxanthine | 56.66 | 15.28 | 3.87 | ✓ | ✓ |  |
| HMDB0000092 | dimethylglycine | 55.98 | 2.25 | ／ | ✓ | ✓ |  |
| HMDB0001453 | thiocyanate | 55.30 | 31.83 | 27.60 |  |  |  |
| HMDB0002500 | bromide | 50.56 | 50.00 | 29.00 |  |  |  |
| HMDB0000517 | l-arginine | 49.93 | 108.42 | 18.21 | ✓ | ✓ |  |
| HMDB0000209 | phenylacetic acid | 49.90 | ／ | ／ | ✓ | ✓ |  |
| HMDB0000033 | carnosine | 40.18 | 3.29 | ／ | ✓ | ✓ | ✓ |
| HMDB0000883 | l-valine | 39.45 | 235.04 | 18.00 | ✓ | ✓ |  |
| HMDB0000056 | beta-alanine | 38.26 | 3.40 | 0.02 |  |  |  |
| HMDB0000214 | ornithine | 35.52 | 76.86 | 4.29 | ✓ | ✓ |  |
| HMDB0000237 | propionic acid | 35.51 | 0.90 | 2.80 |  |  |  |
| HMDB0000172 | l-isoleucine | 35.32 | 67.55 | 7.13 | ✓ | ✓ |  |
| HMDB0000112 | gamma-aminobutyric acid | 34.79 | 0.11 | 0.22 |  |  |  |
| HMDB0000008 | 2-hydroxybutyric acid | 33.59 | 42.23 | 37.50 | ✓ | ✓ | ✓ |
| HMDB0003157 | guanidinosuccinic acid | 32.87 | 0.32 | 0.09 |  |  |  |
| HMDB0000292 | xanthine | 31.19 | 2.28 | 5.20 |  |  |  |
| HMDB0000812 | n-acetyl-l-aspartic acid | 26.39 | ／ | 0.81 | ✓ | ✓ |  |
| HMDB0000156 | malic acid | 23.99 | 7.60 | 3.00 | ✓ | ✓ |  |
| HMDB0000162 | l-proline | 19.62 | 192.92 | ／ | ✓ | ✓ | ✓ |
| HMDB0000696 | l-methionine | 18.60 | 26.93 | 2.96 | ✓ | ✓ | ✓ |
| HMDB0000134 | fumaric acid | 18.11 | 1.50 | 33.00 | ✓ | ✓ |  |
| HMDB0000210 | pantothenic acid | 17.99 | 2.98 | 3.25 |  |  |  |
| HMDB0003229 | palmitoleic acid | 16.97 | 11.73 | 2.00 |  |  |  |
| HMDB0011635 | p-cresol sulfate | 15.59 | ／ | ／ | ✓ | ✓ |  |
| HMDB0000169 | d-mannose | 14.99 | 39.00 | 44.00 |  |  |  |
| HMDB0000014 | deoxycytidine | 7.80 | 0.20 | ／ | ✓ | ✓ | ✓ |
| HMDB0000101 | 2-deoxyadenosine | 2.64 | ／ | ／ | ✓ | ✓ |  |
| HMDB0000676 | l-homocystine | 2.04 | ／ | ／ | ✓ |  |  |
| HMDB0000125 | glutathione | 0.36 | 280.40 | ／ | ✓ | ✓ |  |
| HMDB0000673 | linoleic acid | 0.60 | 215.08 | ／ | ✓ | ✓ |  |
| HMDB0002017 | 1-phenylethylamine | 0.08 | 0.01 | 0.14 |  |  |  |
| HMDB0000121 | folic acid | 0.02 | 0.02 | 0.05 |  |  |  |
| HMDB0003345 | alpha-d-glucose | ／ | 98.00 | ／ | ✓ | ✓ | ✓ |
| HMDB0000131 | glycerol | ／ | 247.96 | 26.25 | ✓ | ✓ |  |
| HMDB0001051 | glyceraldehyde | ／ | 1476.00 | ／ | ✓ | ✓ |  |

- 1. **Experimental procedures for standards**

Solutions of pure compound standards were prepared by dissolving different amounts of the reagents in LC-MS quality water. NMR samples were prepared following the urine SOP described in Dona et al 2014 (Dona et al. 2014). In brief, 540 mL of the pure compound solution ware mixed in a 5mm NMR tube with 60 mL of 1.5 M of KH_2_PO_4_ buffer containing 5.8 mM of TSP (3-(trimethylsilyl)-2,2,3,3-tetradeuteropropionic acid or TMSP-d4) and 2 mM of NaN_3_ prepared in D_2_O at pH 7.4.

For each sample, 1D-NOESY presat pulse sequence and J-Res 2D experiments were acquired in automation at 300 K in a Bruker Avance III HD 600 spectrometer working at 14.1 T equipped with a BBI probe. Each 1D was acquired with 32 Free Induction Decays (FID), 64 K points with a 20-ppm window centered at 4.78 ppm (position of the water proton resonance) while 2D J-Resolved experiments were acquired with 2 FIDs, 8K points and 40 transients. Relaxation delay was set at 4 and 2 s respectively, and a water pre-saturation pulse was applied during this period to cancel the water signal. A 0.3 Hz line-broadening was applied to the 1D-spectrum free induction decay before applying Fourier transform. Topspin 3.6 (Bruker Corporation, Germany) was used for spectrum processing including Fourier transformation, phasing, baseline correction and calibration.

- 1. **Inter-metabolite correlations**

In metabolic profiling, it is essential to consider significant linear correlations between metabolite pairs. To simulate these inter-metabolite correlations, metabolite concentrations are generated by drawing from a multivariate normal distribution. To ensure that these concentrations remain non-negative, rejection sampling is employed. Following the methodology described in (Higham 2002), the nearest positive semidefinite correlation matrix is derived based on the specified pairwise correlations. This matrix is then adjusted by constructing a covariance matrix that incorporates the metabolite standard deviations and the specified correlations. Adjustments are made to the diagonal entries of the covariance matrix to guarantee its positive definiteness.

- 1. **Peak Pre-processing for 1D NMR Spectra**

Before simulating spectra for mixtures, it is crucial to pre-process the spectra of pure compounds to ensure they integrate effectively into the final metabolic profiles.

For 1D ^1^H NMR simulations, pre-processing includes removing calibration peaks and water suppression residual, baseline correction to prevent background noise from distorting metabolite signals, and signal smoothing to reduce noise and prevent artefacts. Additionally, normalization is applied to standardize spectrum intensity, facilitating comparative quantitative analysis. While most steps adhere to the methods outlined in MetAssimulo 1.0, the smoothing process has been refined to better preserve the shapes of spectral signals.

In MetAssimulo 1.0, noise reduction for each pure compound spectrum was achieved using a traditional kernel smoothing approach, with the default kernel type set to “Normal”. In contrast, MetAssimulo 2.0 adopts a more sophisticated method based on centered moving averages to smooth noisy spectral data. In this step, only spectral intensities that fall below a user-defined threshold – defaulting to 10% of the maximum intensity – are targeted for smoothing. This new approach deviates from the conventional use of fixed window sizes. Instead, it employs variable window sizes that adjust dynamically along the spectrum. Specifically, the window size is reduced at points where the original spectral signal intersects with noisy signals and is increased elsewhere along the spectrum. This strategy allows for the preservation of the shapes of spectral signals while primarily capturing the general trend of the noise, enhancing the clarity and utility of the spectral data. The specifics of this advanced smoothing algorithm are detailed below:


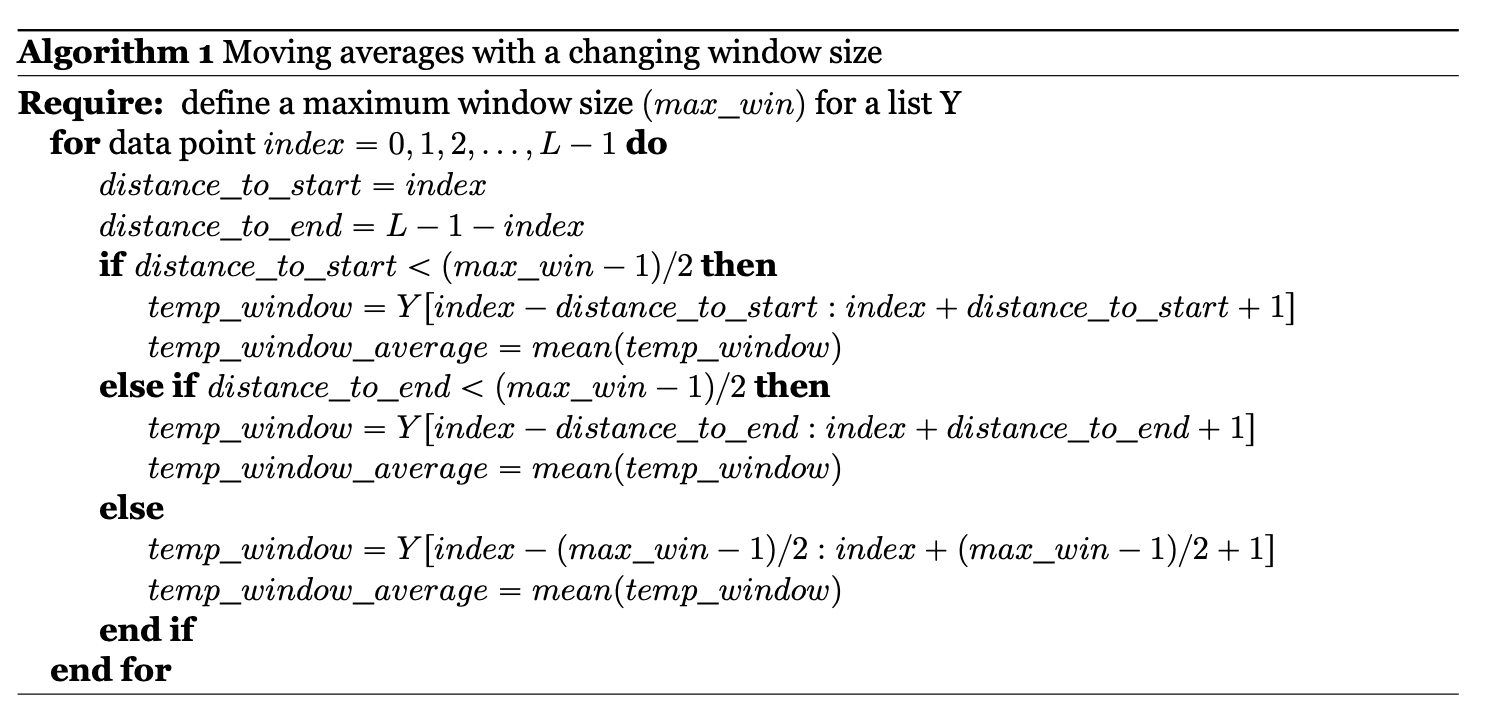


- 1. **Peak Pre-processing for 2D NMR Spectra**

For 2D J-Res and COSY NMR simulations, pre-processing also involves removing calibration peaks and water residuals. Specific to 2D data, noise is reduced to a predetermined threshold, and smoothing is performed using the ‘convolve2d’ function from the SciPy package. Normalization adjusts the intensity values to a scale from 0 to 1, enhancing both the clarity and accuracy of the spectral data.

- 1. **Peak detection for 1D NMR spectra**

MetAssimulo 2.0 exploits a simpler and more efficient approach for peak detection in one-dimensional ^1^H NMR spectral data as outlined in Algorithm 2. Initially, it detects peaks within the spectrum by applying the $find\_peaks$ function from $scipy$ package, which isolates peaks based on a user-defined threshold relative to the maximum signal intensity. Following peak detection, the algorithm generates a binary signal mask by comparing each data point against the mean value of the spectrum, classifying points as ‘1’ if they exceed the mean and ‘0’ otherwise. This binary mask effectively segments the spectrum into potential regions of interest by grouping consecutive ‘1’s into continuous segments, identifying these as possible clusters. Each of these segments is then examined to determine if it contains any of the previously detected peaks. Only those segments that include at least one peak are retained as valid peak clusters. This method ensures that the final output consists solely of significant clusters, potentially corresponding to meaningful spectral features, thereby enhancing the analytical utility of the NMR data.


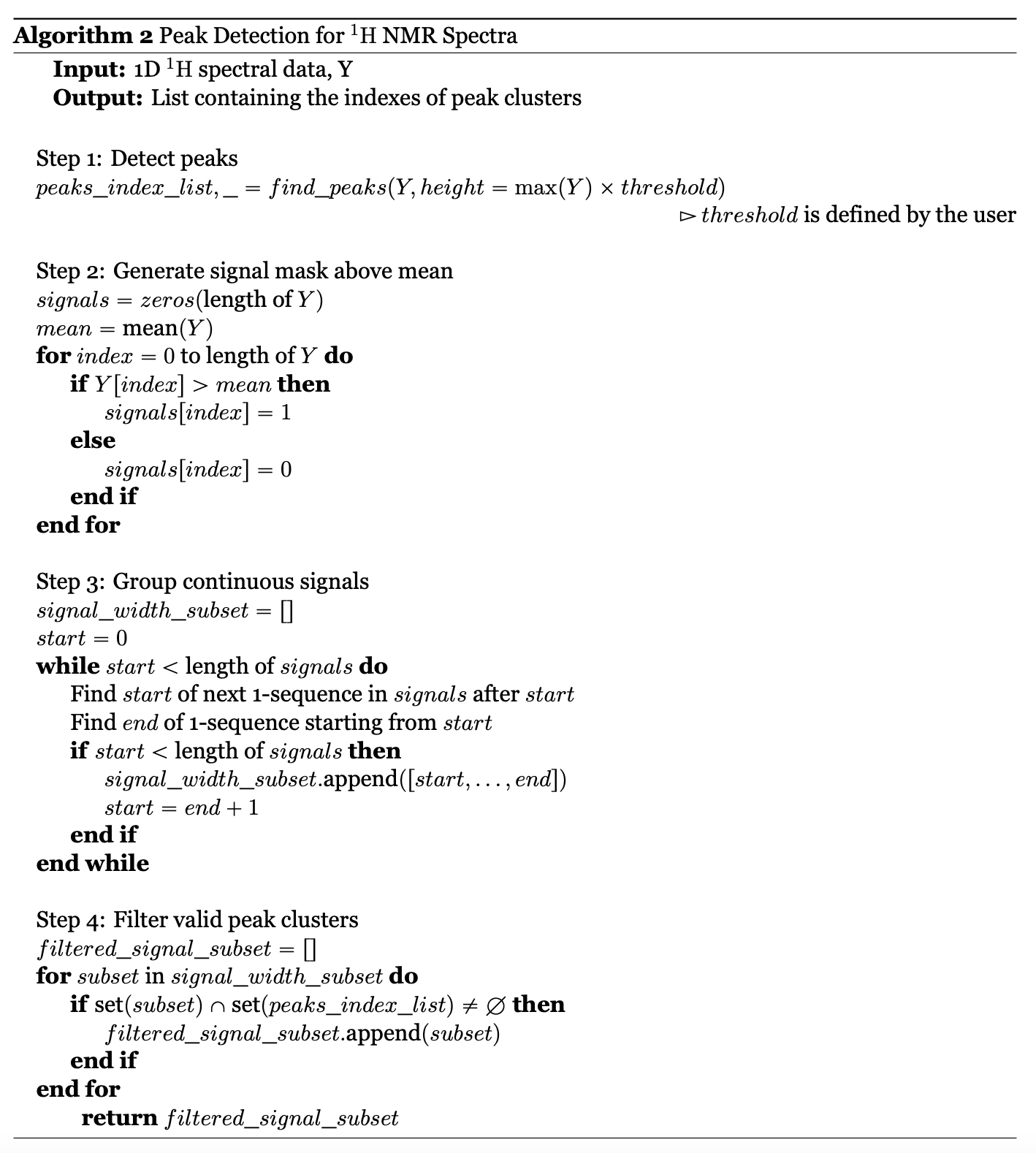


- 1. **Derivation of the transformation of the Henderson-Hasselbalch Equation**

In the analysis, the observed chemical shift $\delta_{obs}$ is modeled using Henderson-Hasselbalch equation (Ackerman et al. 1996) as detailed in Equation (1). This equation integrates the acid and base limiting chemical shifts, $\delta_{L}$ and $\delta_{HL}$, respectively, alongside the metabolite’s pKa and the solution’s pH. Through a series of transformations from Equation (1a) to Equation (1e), Equation (1) is refined into Equation (2), which offers a simplified and more interpretable form.

Considering a single resonance from a metabolite in two distinct pH environments, ${pH}_{0}$ and ${pH}_{1}$, its chemical shifts are captured in Equation (3) for ${pH}_{0}$ and Equation (4) for ${pH}_{1}$. The differential chemical shift between these environments is quantified in Equation (5a), representing the peak shift. Subsequent derivation leads to Equation (5b), which serves as the final expression derived from the Henderson-Hasselbalch equation, effectively illustrating the relationship between pH and chemical shift for the resonance.

$\boldsymbol{\delta}_{\boldsymbol{obs}}\boldsymbol{=}\frac{\boldsymbol{\delta}_{\boldsymbol{L}}\boldsymbol{+}\boldsymbol{\delta}_{\boldsymbol{HL}}\boldsymbol{(}\boldsymbol{10}^{\boldsymbol{(pH-pKa)}}\boldsymbol{)}}{\boldsymbol{1+}\boldsymbol{10}^{\boldsymbol{(pH-pKa)}}}$ **(1)**

$\left( 1+{10}^{\left( pH-pKa \right)} \right)\delta_{obs}=\delta_{L}+\delta_{HL}\left( {10}^{\left( pH-pKa \right)} \right)$ (1a)

${{10}^{\left( pH-pKa \right)} \times(\delta}_{obs}-\delta_{HL})=\delta_{L}-\delta_{obs}$ (1b)

${10}^{\left( pH-pKa \right)}=\frac{\delta_{L}-\delta_{obs}}{{\delta_{obs}-\delta}_{HL}}$ (1c)

$1+{10}^{\left( pH-pKa \right)}=\frac{\delta_{L}-\delta_{obs}+{\delta_{obs}-\delta}_{HL}}{{\delta_{obs}-\delta}_{HL}}$ (1d)

$1+{10}^{\left( pH-pKa \right)}=\frac{\delta_{L}-\delta_{HL}}{{\delta_{obs}-\delta}_{HL}}$ (1e)

$\boldsymbol{\delta}_{\boldsymbol{obs}}\boldsymbol{=}\frac{\boldsymbol{\delta}_{\boldsymbol{L}}\boldsymbol{-}\boldsymbol{\delta}_{\boldsymbol{HL}}}{\boldsymbol{1}\boldsymbol{+}\boldsymbol{10}^{\left( \boldsymbol{pH}\boldsymbol{-}\boldsymbol{pKa} \right)}}\boldsymbol{+}\boldsymbol{\delta}_{\boldsymbol{HL}}$ **(2)**

$\delta_{obs0}=\frac{\delta_{L}-\delta_{HL}}{1+{10}^{\left( {pH}_{0}-pKa \right)}}+\delta_{HL}$ (3)

$\delta_{obs1}=\frac{\delta_{L}-\delta_{HL}}{1+{10}^{\left( {pH}_{1}-pKa \right)}}+\delta_{HL}$ (4)

$\Delta\delta=\delta_{obs1}-\delta_{obs0}=\frac{\delta_{L}-\delta_{HL}}{1+{10}^{\left( {pH}_{1}-pKa \right)}}- \frac{\delta_{L}-\delta_{HL}}{1+{10}^{\left( {pH}_{0}-pKa \right)}}$ (5a)

$\boldsymbol{\Delta}\boldsymbol{\delta}\boldsymbol{=}\frac{\left( \boldsymbol{\delta}_{\boldsymbol{L}}\boldsymbol{-}\boldsymbol{\delta}_{\boldsymbol{HL}} \right)\boldsymbol{\times}\left( \boldsymbol{10}^{\boldsymbol{pH}_{\boldsymbol{0}}\boldsymbol{-}\boldsymbol{pKa}}\boldsymbol{-}\boldsymbol{10}^{\boldsymbol{pH}_{\boldsymbol{1}}\boldsymbol{-}\boldsymbol{pKa}} \right)}{\left( \boldsymbol{1}\boldsymbol{+}\boldsymbol{10}^{\boldsymbol{pH}_{\boldsymbol{0}}\boldsymbol{-}\boldsymbol{pKa}} \right)\boldsymbol{\times}\left( \boldsymbol{1}\boldsymbol{+}\boldsymbol{10}^{\boldsymbol{pH}_{\boldsymbol{1}}\boldsymbol{-}\boldsymbol{pKa}} \right)}$ **(5b)**

- 1. **Peak shift for 2D NMR spectra**

In the processing of 2D J-Res NMR spectra, peak shifting is straightforwardly implemented by shifting positions along the F2 axis. This method of peak shifting in J-Res spectra parallels the techniques employed for 1D NMR spectra, where shifts are similarly constrained to a single dimension.

Conversely, the process for 2D COSY NMR spectra is more complex due to the necessity of shifting peaks along both the F1 and F2 dimensions. This bidimensional adjustment requires a more intricate approach to accurately align peaks within the two-dimensional spectral plane. The specifics of this method are comprehensively detailed in Algorithm 3, which provides a structured framework for implementing peak shifts in 2D COSY spectra, ensuring precise modifications to the spectral data.


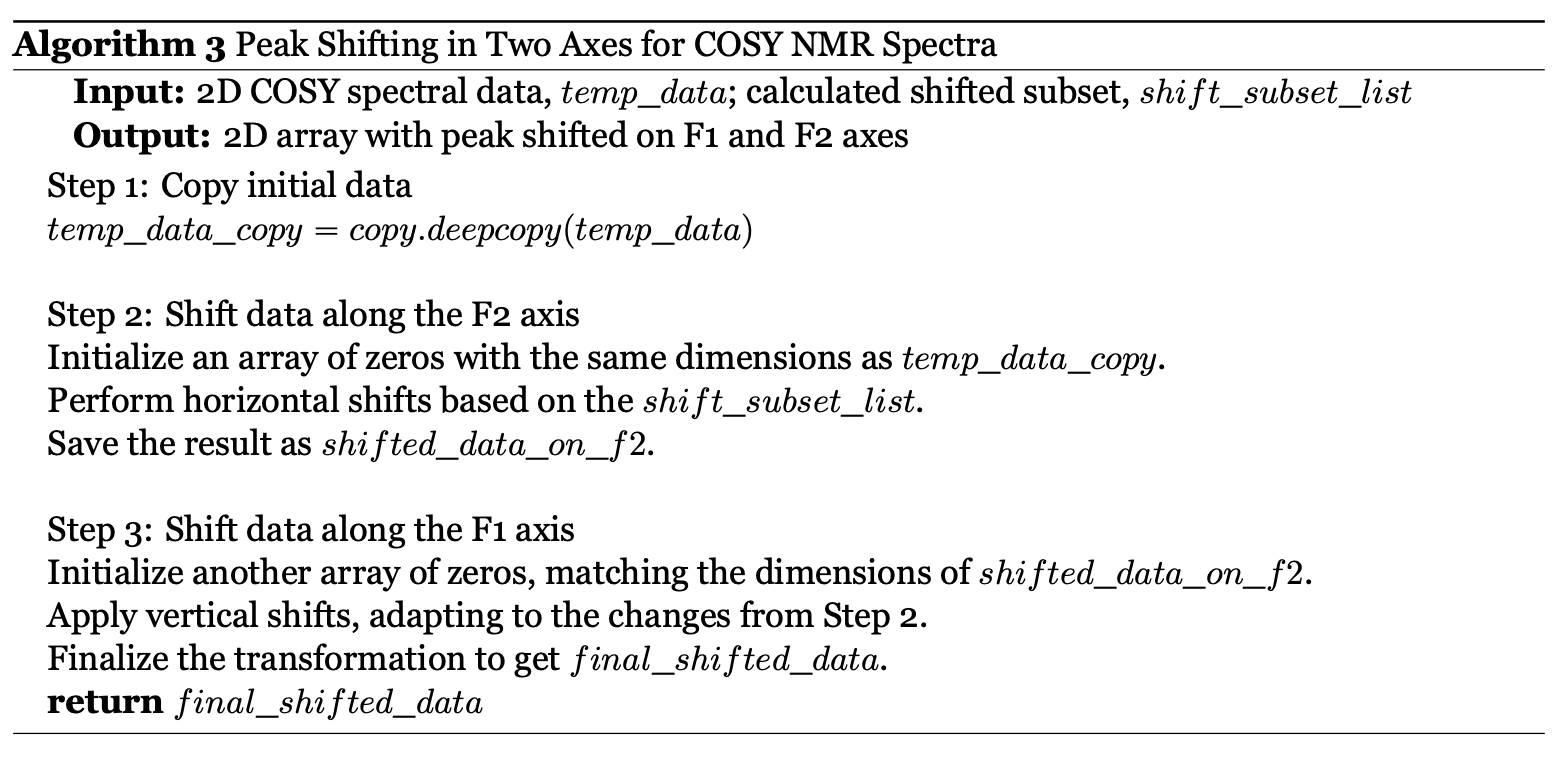


1. **Results**
   1. **Workflow of MetAssimulo 2.0**

MetAssimulo 2.0 features a structured interface including a home page and three specialized pages designed for simulating 1D ^1^H NMR spectra, 2D J-Res NMR spectra, and 2D COSY NMR spectra, respectively. The workflows across these pages are uniform and delineated into three primary steps, as illustrated in Figure S1.

***Selection of Metabolites***: Initially, users select metabolites from the local database, as detailed in Table S1. These selected metabolites are then cross-referenced with the HMDB (Wishart et al. 2022) to acquire corresponding HMDB identifiers. This linkage facilitates the retrieval of accurate information concerning the metabolites’ concentrations, pKa values, and proton counts, ensuring the simulations are based on precise biochemical data.

***Concentration Simulation***: The second step offers two options for simulating concentrations. Users have the flexibility to either upload their own concentration data or modify concentration tables that have been aggregated from HMDB data. This adaptability allows users to tailor the simulation parameters to specific research needs or experimental conditions.

***Spectrum Simulation***: In the final step, users can simulate the mixture spectra. This simulation can be conducted either without peak shifts or with peak shifts, depending on the users’ requirements. Additionally, the pH value for each replicate can be adjusted by the user, providing further control over the simulation conditions and enhancing the authenticity of the spectral output.

This structured workflow ensures that MetAssimulo 2.0 provides a comprehensive and user-friendly environment for the simulation of NMR spectra, accommodating a wide range of scientific inquiries and experimental scenarios.


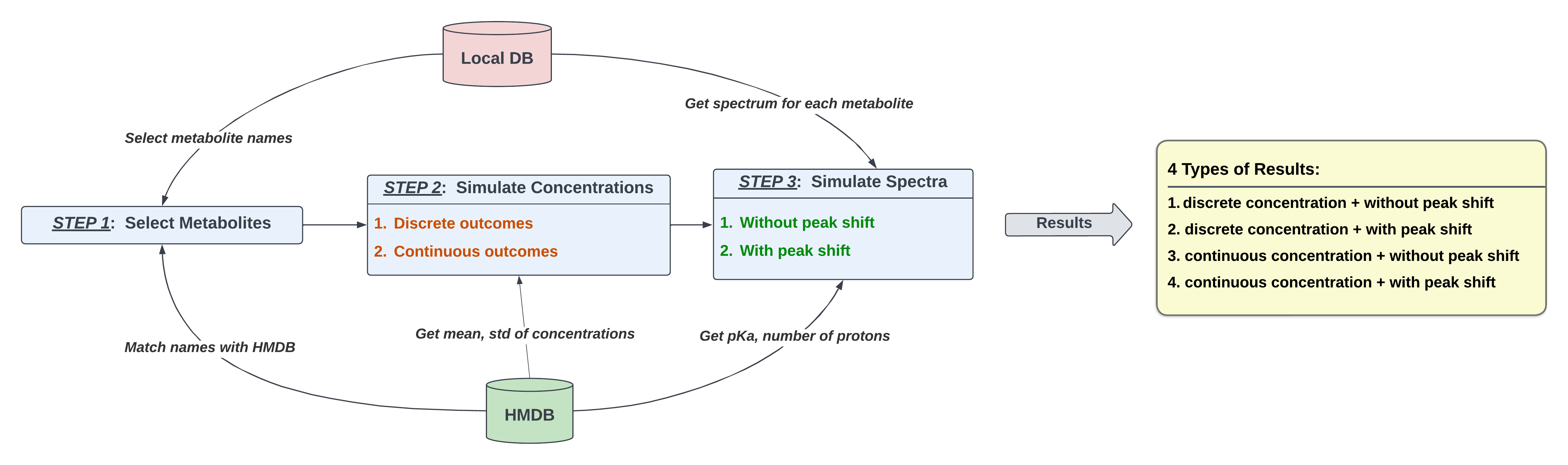


**Figure S1.** The workflow of MetAssimulo 2.0 web application

- 1. **Peak shifting in 1D NMR Spectra**


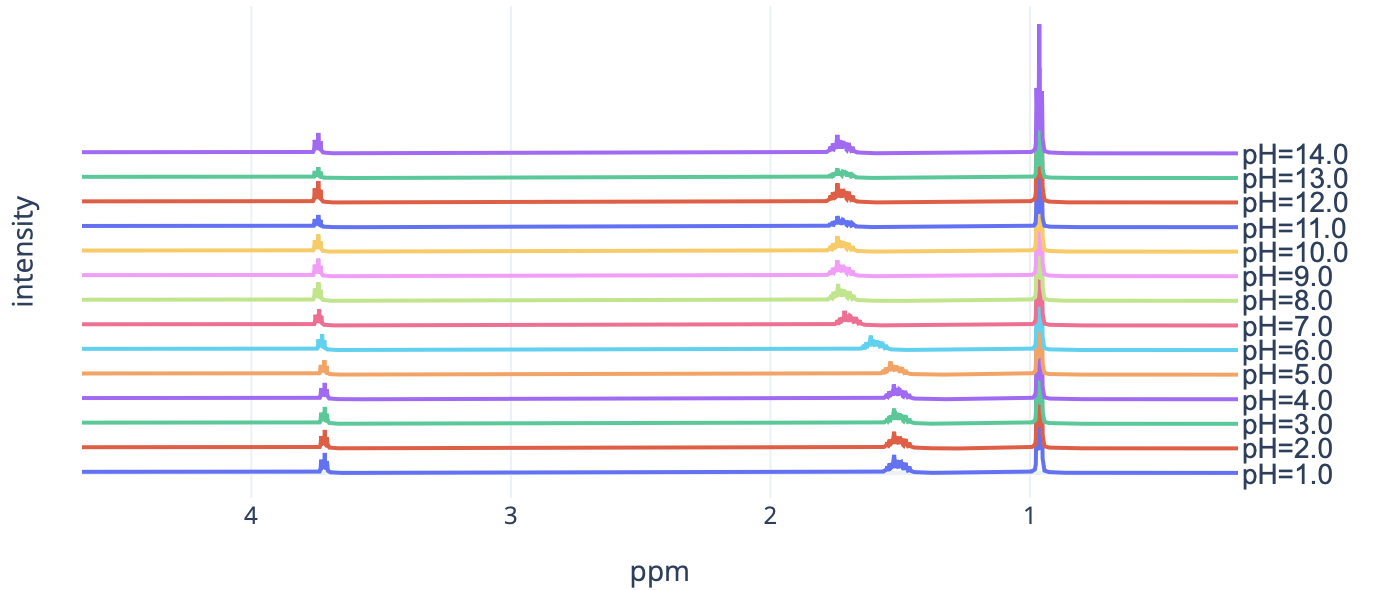


**Figure S2.** Peak Shift Variations in Leucine Across Different pH Levels. This figure illustrates the positional changes of leucine peaks on ppm scales in various pH environments. The peak cluster around 1.5 ppm exhibits the most significant shift across different pH values, followed by the cluster around 3.7 ppm. The cluster near 1 ppm shows minimal variation. The observed trends in peak shifts for leucine conform to the sigmoidal pattern described in Equation (3) of the main manuscript.

- 1. **Simulated COSY Spectrum for Urine Mixtures**


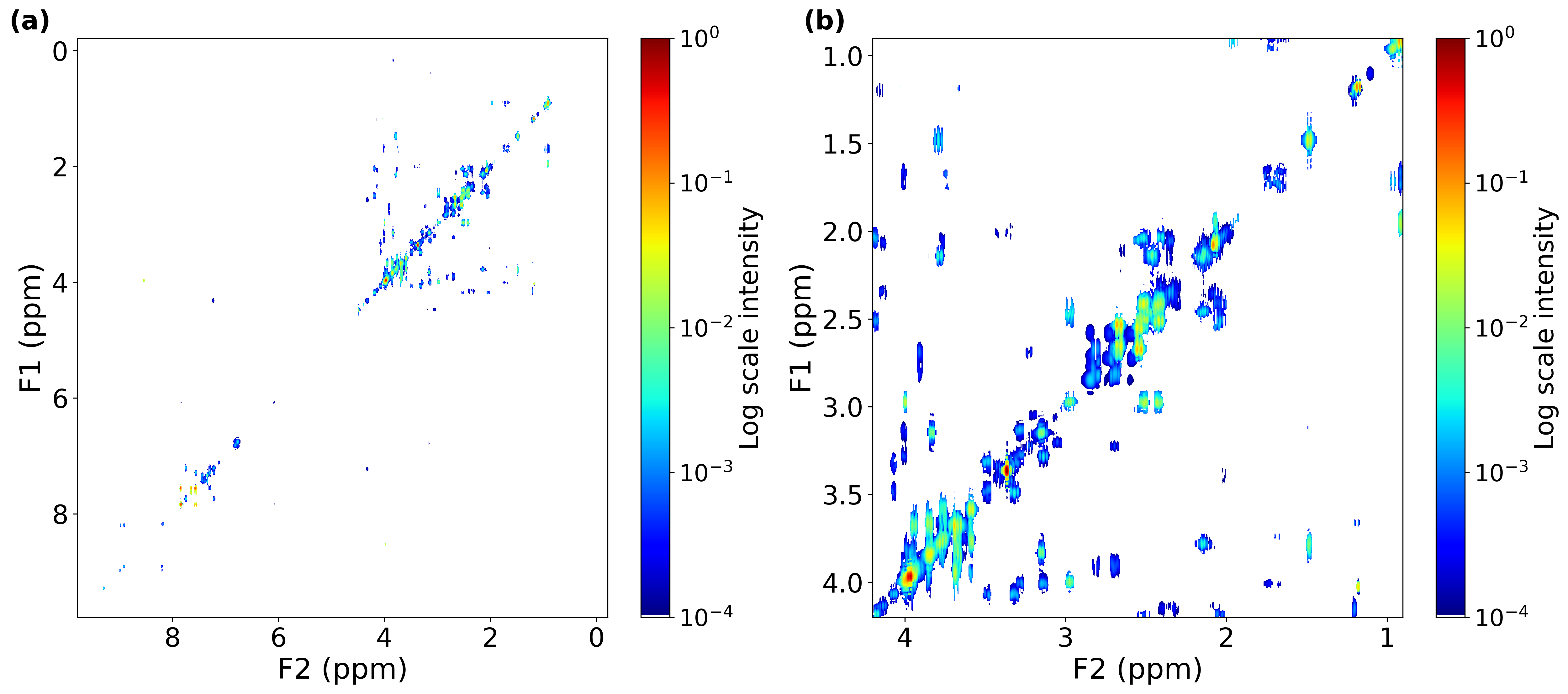


**Figure S3.** Simulated 2D COSY NMR Spectrum for Human Urine Mixtures. Panel (a) displays the spectrum generated by MetAssimulo 2.0, incorporates the 24 metabolites with available COSY spectra, as listed in Table S1, which are frequently detected in human urine. The spectrum is normalized to a maximum intensity of one and is presented with a logarithmic color scale on a white background to enhance visibility. Panel (b) shows a detailed view of the spectrum from 1-4 ppm.

- 1. **Table for metabolites used for continuous outcome simulation (BMI)**

**Table S2.** Metabolite Information Used for Simulating Blood Profiles Associated with BMI. This table lists 10 metabolites, providing their names, HMDB IDs, and mean and standard deviation (Std) of concentrations in blood extracted from HMDB (Wishart et al. 2022). Additionally, it includes the correlation coefficient (a) with BMI, sourced from (Moore et al. 2014). Among these, 10 metabolites show a correlation with BMI, for which the standard deviation of random error is consistently defined as 0.2.

| Name | HMDB ID | Mean (uM) | Std (uM) | Slope, a | Std of random error |
| --- | --- | --- | --- | --- | --- |
| glycine | HMDB0000123 | 275.95 | 66.22 | -0.21 | 0.2 |
| glycerol | HMDB0000131 | 247.96 | 73.82 | 0.14 | 0.2 |
| l-valine | HMDB0000883 | 235.04 | 41.70 | 0.25 | 0.2 |
| l-leucine | HMDB0000687 | 124.20 | 16.46 | 0.22 | 0.2 |
| histidine | HMDB0000177 | 99.85 | 17.25 | -0.21 | 0.2 |
| l-tyrosine | HMDB0000158 | 83.43 | 10.75 | 0.21 | 0.2 |
| l-lactate | HMDB0000190 | 2011.80 | 734.29 | 0.12 | 0.2 |
| l-phenylalanine | HMDB0000159 | 77.72 | 9.30 | 0.16 | 0.2 |
| l-isoleucine | HMDB0000172 | 67.55 | 12.17 | 0.22 | 0.2 |
| l-asparagine | HMDB0000168 | 51.56 | 8.08 | -0.19 | 0.2 |

- 1. **Full 2D J-Res Spectra of Simulated and Real urine mixtures**


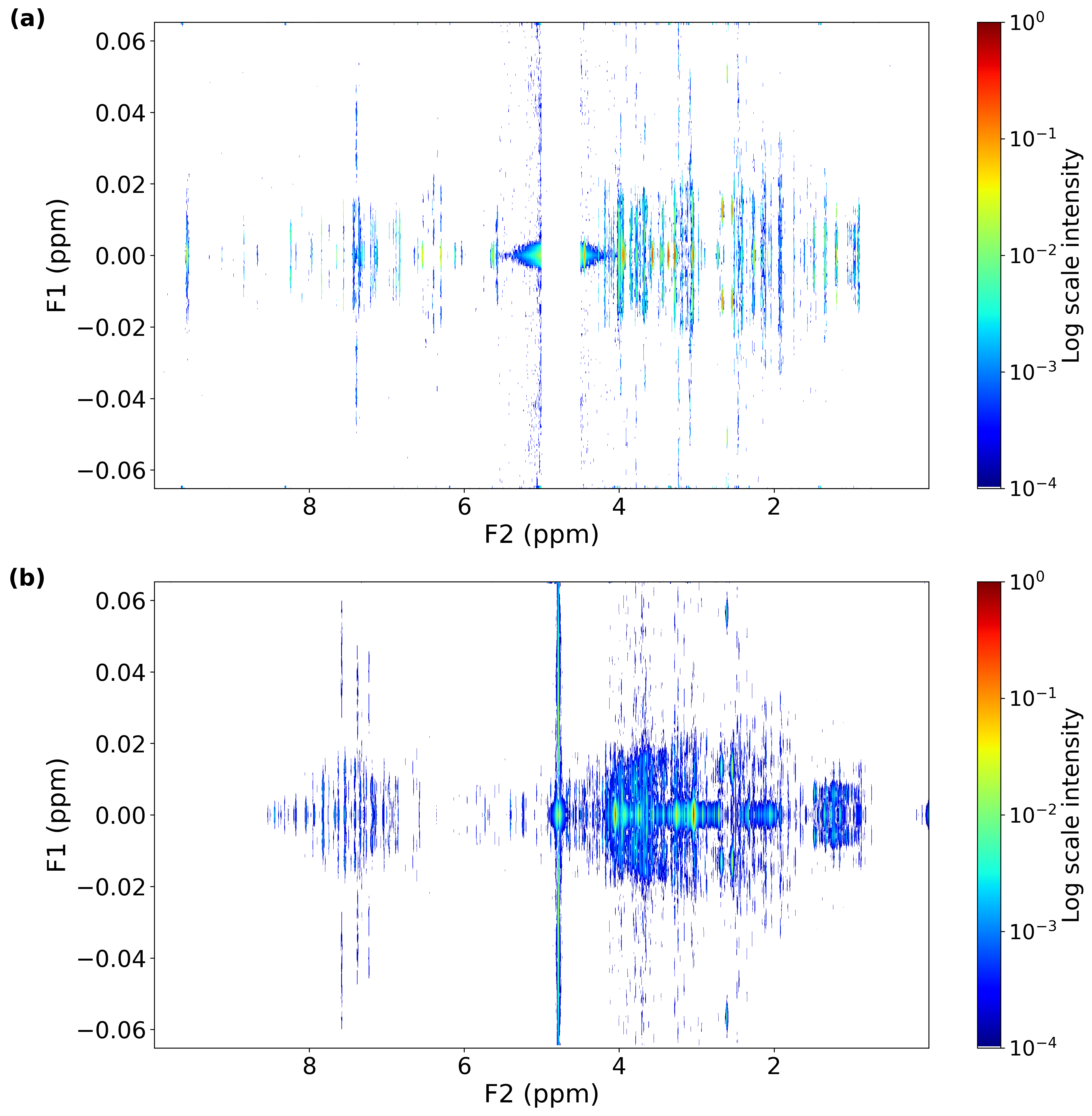


**Figure S4.** (a) simulated 2D J-Res urine spectrum from 0 to 10 ppm. (b) real 2D J-Res urine spectrum from 0 to 10 ppm.

- 1. **Comparison with MetAssimulo 1.0**

Table S3 provides a detailed comparison between MetAssimulo 2.0 and MetAssimulo 1.0, highlighting several key enhancements that elevate the utility and accessibility of the newer version. MetAssimulo 2.0, developed as an easily accessible web application, markedly improves user accessibility compared to the desktop-based original. It supports a wider selection of metabolites, offering 75 compared to the original 48, thus broadening the scope of potential simulations. Additionally, while the MetAssimulo 1.0 was limited to simulating human urine, MetAssimulo 2.0 extends its capabilities to include a diverse array of biospecimens such as urine, blood, and cerebrospinal fluid (CSF), thereby accommodating more comprehensive biomedical investigations.

Another significant enhancement in MetAssimulo 2.0 is the ability to simulate continuous outcome results, which is crucial for exploring the relationships between various biological conditions and metabolite profiles. Furthermore, MetAssimulo 2.0 can simulate both 2D J-Res and COSY spectral data for complex mixtures, which would facilitate the process of metabolite annotation or identification, especially in complex biofluids. The implementation of an improved algorithm for peak detection also ensures the generation of smoother and more accurate NMR spectra, enhancing the reliability of spectral data analysis. These advancements make MetAssimulo 2.0 a robust and versatile tool for a wide range of scientific applications in the field of bioinformatics.

Additionally, Table S3 also presents a comparison between MetAssimulo 2.0 and GISSMO (Dashti et al. 2017) which is designed for simulating 1D ^1^H NMR spectra of metabolites across different field strengths, and it also includes a feature that allows the generation of simple mixture spectra. A key distinction between MetAssimulo 2.0 and GISSMO in mixture spectra simulation is that MetAssimulo 2.0 simulates mixture spectra using experimental data for pure compounds, whereas GISSMO relies on simulated spectra for pure compounds. Moreover, GISSMO lacks support for 2D NMR spectra simulation of mixtures. A more detailed comparison can be found in the table below.

**Table S3. Comparison between MetAssimulo 2.0 and MetAssimulo 1.0.**

|  | MetAssimulo 2.0 | MetAssimulo 1.0 | GISSMO |
| --- | --- | --- | --- |
| Simulator Type | Python-based web app | MATLAB-based package | Software package |
| No of metabolites | 75 | 48 | More than 400 simulated compounds |
| Inter-metabolite correlations | ✓ | ✓ | 🗶 |
| Urine | ✓ | ✓ | ／^*^ |
| Blood | ✓ | 🗶 | ／^*^ |
| CSF | ✓ | 🗶 | ／^*^ |
| Discrete outcomes | ✓ | ✓ | 🗶 |
| Continuous outcomes | ✓ | 🗶 | 🗶 |
| 1D ^1^H NMR spectra | ✓ | ✓ | ✓ |
| 2D J-Res NMR spectra | ✓ | 🗶 | 🗶 |
| 2D COSY NMR spectra | ✓ | 🗶 | 🗶 |
| With peak shifts | ✓ | ✓ | ✓ |
| Peak shifting methods | Peak cluster detection (section 1.5 & 1.6) | Peak cluster cross-referenced with HMDB | By tuning spin system matrices |
| At any field strength | 🗶 | 🗶 | ✓ |

^*^: GISSMO does not provide any concentration information for any specific biofluids, but users can upload their own concentration data to simulate different biofluids.

**Reference**

Ackerman, Joseph J.H., Soto, Gabriel E., Spees, William M., Zhu, Zehua, and Evelhoch, Jeffrey L., ‘The NMR Chemical Shift PH Measurement Revisited: Analysis of Error and Modeling of a PH Dependent Reference’, *Magnetic Resonance in Medicine*, 36/5 (1996)

Dashti, Hesam, Westler, William M., Tonelli, Marco, Wedell, Jonathan R., Markley, John L., and Eghbalnia, Hamid R., ‘Spin System Modeling of Nuclear Magnetic Resonance Spectra for Applications in Metabolomics and Small Molecule Screening’, *Analytical Chemistry*, 89/22 (2017)

Dona, Anthony C., Jiménez, Beatriz, Schafer, Hartmut, Humpfer, Eberhard, Spraul, Manfred, Lewis, Matthew R., et al., ‘Precision High-Throughput Proton NMR Spectroscopy of Human Urine, Serum, and Plasma for Large-Scale Metabolic Phenotyping’, *Analytical Chemistry*, 86/19 (2014)

Higham, Nicholas J., ‘Computing the Nearest Correlation Matrix - A Problem from Finance’, *IMA Journal of Numerical Analysis*, 22/3 (2002)

Moore, Steven C., Matthews, Charles E., Sampson, Joshua N., Stolzenberg-Solomon, Rachael Z., Zheng, Wei, Cai, Qiuyin, et al., ‘Human Metabolic Correlates of Body Mass Index’, *Metabolomics*, 10/2 (2014)

Wishart, David S., Guo, An Chi, Oler, Eponine, Wang, Fei, Anjum, Afia, Peters, Harrison, et al., ‘HMDB 5.0: The Human Metabolome Database for 2022’, *Nucleic Acids Research*, 50/D1 (2022)
